# A fine pore-preserved deep neural network for porosity analytics of a high burnup U-10Zr metallic fuel

**DOI:** 10.1038/s41598-023-48800-3

**Published:** 2023-12-14

**Authors:** Haotian Wang, Fei Xu, Lu Cai, Daniele Salvato, Fidelma Giulia Di Lemma, Luca Capriotti, Tiankai Yao, Min Xian

**Affiliations:** 1 University of Idaho, Idaho Falls, ID USA; 2 Idaho National Laboratory, Idaho Falls, ID USA

**Keywords:** Materials science, Nuclear energy

## Abstract

U-10 wt.% Zr (U-10Zr) metallic fuel is the leading candidate for next-generation sodium-cooled fast reactors. Porosity is one of the most important factors that impacts the performance of U-10Zr metallic fuel. The pores generated by the fission gas accumulation can lead to changes in thermal conductivity, fuel swelling, Fuel-Cladding Chemical Interaction (FCCI) and Fuel-Cladding Mechanical Interaction (FCMI). Therefore, it is crucial to accurately segment and analyze porosity to understand the U-10Zr fuel system to design future fast reactors. To address the above issues, we introduce a workflow to process and analyze multi-source Scanning Electron Microscope (SEM) image data. Moreover, an encoder-decoder-based, deep fully convolutional network is proposed to segment pores accurately by integrating the residual unit and the densely-connected units. Two SEM 250 × field of view image datasets with different formats are utilized to evaluate the new proposed model’s performance. Sufficient comparison results demonstrate that our method quantitatively outperforms two popular deep fully convolutional networks. Furthermore, we conducted experiments on the third SEM 2500 × field of view image dataset, and the transfer learning results show the potential capability to transfer the knowledge from low-magnification images to high-magnification images. Finally, we use a pre-trained network to predict the pores of SEM images in the whole cross-sectional image and obtain quantitative porosity analysis. Our findings will guide the SEM microscopy data collection efficiently, provide a mechanistic understanding of the U-10Zr fuel system and bridge the gap between advanced characterization to fuel system design.

## Introduction

Early experimental fast reactors, such as Experimental Breeder Reactor I (EBR-I), and Experimental Breeder Reactor II (EBR-II), provided a large pool of irradiated experiments demonstrating the high performance, safety, and possibility of close fuel cycle using metallic fuel in sodium fast reactors (SFR). Further studies in the Fast Flux Test Facility (FFTF) and the Transient Reactor Test Facility (TREAT) provide further information on various fuel designs and materials, further demonstrating the possibility of achieving higher core performance^1–6^.

A limiting fuel performance phenomenon for this fuel system is irradiation gas swelling that leads to an increase in Fuel-Cladding Chemical Interaction (FCCI) and Fuel-Cladding Mechanical Interaction (FCMI), which can limit burnup extension. Currently, U–Zr-based metallic fuels are pursued as primary candidate fuels for Generation IV SFRs, due to their high thermal conductivity, high burnup capabilities, and promising neutronic performance. Among them, U-10 wt.% Zr (U-10Zr) metallic fuel, cladded with HT9 alloy, has been tested up to a high burnup of 20 at.%, without cladding breach^7^.

In the last years, over 10,000 U-10Zr fuel pins have been irradiated in the test reactors and new advanced Post-Irradiation Examination (PIE) data has been collected at Idaho National Laboratory (INL). The data including observations on fuel swelling, Zr redistribution, FCCI, and FCMI phenomena, obtained through various characterization techniques such as neutron radiography, pin profilometry, gamma scan, fission gas release analysis, metallography, micro-hardness, isotopic chemical analysis, and high-resolution/micro-nano scale characterization^8–17^. New advanced characterization techniques employ multi-source data, including Scanning Electron Microscopy (SEM) images, Energy-Dispersive Spectroscopy (EDS) images, and Scanning Transmission Electron Microscopy (STEM), which provide qualitative material information from the optical scale, overall microstructure and pore distribution from SEM and EDS microscale, and phase/crystal structure identification from STEM nanoscale.

Advanced PIE technologies have enabled researchers to gain qualitative understanding of the irradiation behavior of U-10Zr fuel, including Zr redistribution, FCMI, and FCCI^8–17^. For example, Salvato et al.^12^ investigated the Zr redistribution on a U-10Zr fuel cross-section, named FA, which was irradiated to a burnup of approximately 13.2 at.%. The study revealed three major Zr concentric zones within the fuel pin (a Zr-rich central region, a Zr-lean intermediate region, and a Zr intermediate peripherical region) and found that the irradiation temperature and time spent in the reactor were the primary factors affecting the formation of redistribution zones and their extension along the fuel cross-section, rather than the fuel burnup. While the existing literature has provided detailed information on the Zr redistribution behavior of the fuel, it is important to note that U-10Zr fuel performance is also impacted by other factors, such as swelling, FCCI, and FCMI.

The evolution of porosity in U-10Zr metallic fuel changes the fuel properties and impacts the heat transport and constituent redistribution phenomena^18^. The accumulation of fission gas in U-10Zr fuel leads to the evolution of porosity, which can impact thermal conductivity, cause fuel swelling, and ultimately affect FCCI and FCMI. It is crucial to quantitatively analyze porosity to gain insights into fuel performance, particularly the distribution of porosity in different Zr concentric zones and its variation along the thermal gradient from the hot fuel center to the cool rim surface.

**Figure 1:**
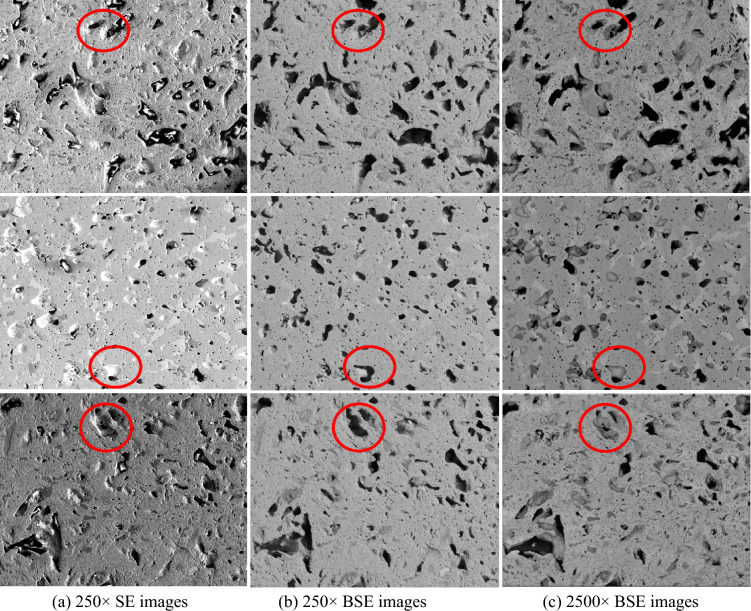
Image patches of U-10Zr fuel from SE and BSE images captured at different magnifications. In each row, red circles highlight the target regions in different image formats and magnification, the same microstructures could have large variance in color and texture appearance.

However, a major challenge remains in porosity analytics. The advanced characterization data collected from different instruments may have different image types, and microscopic length scales, resulting in large variances in the appearance of pores in SEM images. As illustrated in Fig. 1, in the first column, different image patches from various locations have different image conditions but are formatted the same. In each row, the same image patch appears in different image formats, Secondary Electron Microscopy (SE) and Backscattered Electron (BSE) image, or microscopic length scales, 250 × and 2500 × . SE images display the topography of the samples, while BSE images show the composition of the samples. High-magnification images could show more detailed microstructures and pores, particularly on the pore boundaries. The multi-source, multi-scale, and varied image conditions have large morphological variance pores, and large color and texture differences among images, making it challenging to detect and segment pores using traditional image processing techniques, such as image thresholding.

Machine learning algorithms have achieved great success in quantitatively predicting material properties and accelerating materials characterization by integrating physics information, such as loops, cavities, and grain boundary detection on STEM nanoscale images^19–24^. For instance, Xu and Cai et al. developed a Decision Tree-based machine learning model for fission gas pore classification on annular U-10Zr fuels^24,25^; however, the tree-based method is non-robust and sensitive to the SEM images at different formats and length scales. Recently, convolutional neural networks (CNNs) achieved advanced performance and robustness in segmenting, recognizing, and classifying natural and microscopy images^13,26–36^. However, there remain challenges for CNNs to accurately detect pores from the SEM images. 1) CNNs require a large amount of annotated data for converging, however, manually annotating images are expensive, time, and labor consuming. 2) It can be challenging to recognize the morphology of pores in SEM images due to the presence of a large number of small pores. Moreover, the morphology of pores can vary significantly in terms of size and shape, and different microstructures can cluster around the pores. 3) A robust model that can be applied to different types of image formats and various microscopic length scales in practice is needed.

In this study, we proposed a fine pore-preserve deep neural network for robust and accurate pore segmentation which eventually results in more accurate and reliable porosity analysis. In the following section, two major contributions were involved 1) Section "Material and data collection" proposes a data collection and preparation workflow for producing SEM images and their ground truths; and 2) Section "Fine pore-preserving network for pore segmentation" describes the architecture and the learning objective of the proposed deep learning model.

## Proposed method

### Material and data collection

**Figure 2:**
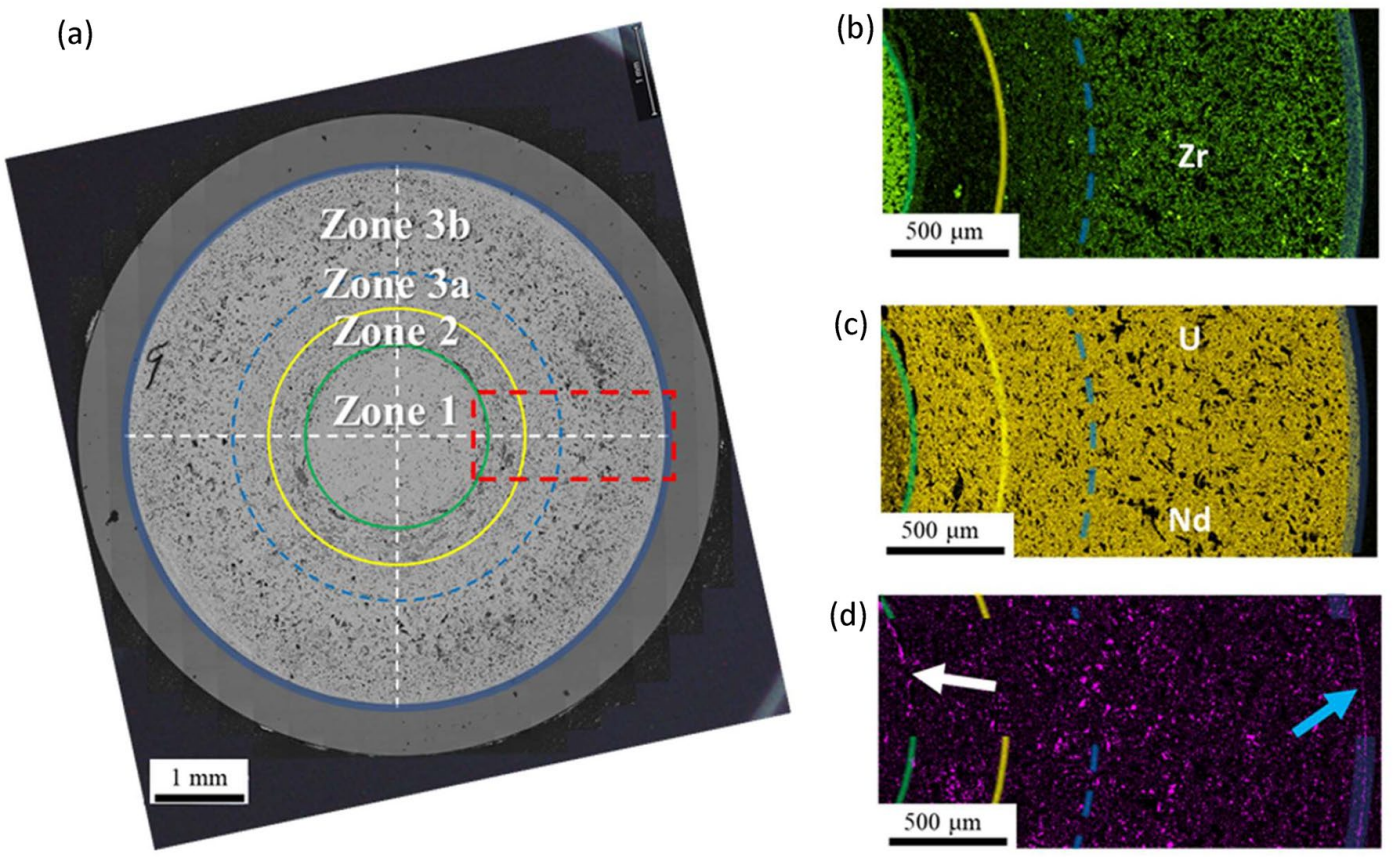
(**a**) A cross-sectional Backscattered Electron (BSE) image at 250 × magnification. (**b**–**d**) Distribution of Zr, U, and Nd, respectively, in the red box region highlighted in (**a**)^12^.

The U-10Zr fuel FA has a diameter of 0.498 cm with three different Zr concentric regions. Zone 1 extends from the cross-section’s center down to 0.32 R, where R represents the pin radius. The fuel center is noted R as 0 and the cladding inner boundary is noted R as 1. On the other hand, Zone 2 develops between 0.32 R and 0.48 R, while Zone 3 between 0.48 R and 1 R, with the boundary between Zone 3a and 3b placed at approximately 0.63 R, as shown in Fig. 2^12^.

Advanced characterization techniques at INL, e.g., focused ion beam (FIB) sampling, scanning transmission electron microscopy (STEM), local thermal conductivity microscopy (TCM), and energy dispersive spectroscopy (EDS), have been applied to the FA sample. FIB cross-section data provides overall fuel appearance, including pores, and different types of microstructures; EDS images show the distributions of chemical elements, and STEM EDS images give the element content percentages for each microstructure. SE images are used to digitalize the surface region of the material. BSE images are used to detect the contrast between areas with different chemical positions from the deeper regions of the image samples. EDS images give the element content percentages for each microstructure. In this study, site-specific lamellae were extracted. Ion trenching, polishing, and lift-out were carried out with a high-energy Xe beam. After that, the thinning and final polishing were done in a FEI Quanta 3D FIB with Ga beams at various energies. We collected the images patches on the cross-section under three different magnifications as shown in Table 1. All the images were collected using a FEI Helios plasma focused ion beam-scanning electron microscopy dual beam (PFIB/SEM) instrument. During cross-section SE/BSE image collecting, the instrument scans the whole sample from left to right, top to bottom, and saves the images one by one. The following image patch will have a 10% overlap with the previous one. However, there is dislocation/drifting existing in the vertical direction which indicates the image patches cannot be catenated directly to get the cross-section one. We utilized ImageJ^37^ software to stitch the image patches into a 250 × whole cross-section according to the coordinate information.

**Table 1:** Advanced characterization data for fuel FA.

	First Polish			Second polish
	50 × (2.85 µm/pixel)	250 × (0.545 µm/pixel)	2500 × (0.0545 µm/pixel)	2500 × (0.0545 µm/pixel)
BSE images	31	166	10,549	10,549
SE images	31	166		
EDS images	4	17		8

#### Data preparation

**Figure 3:**
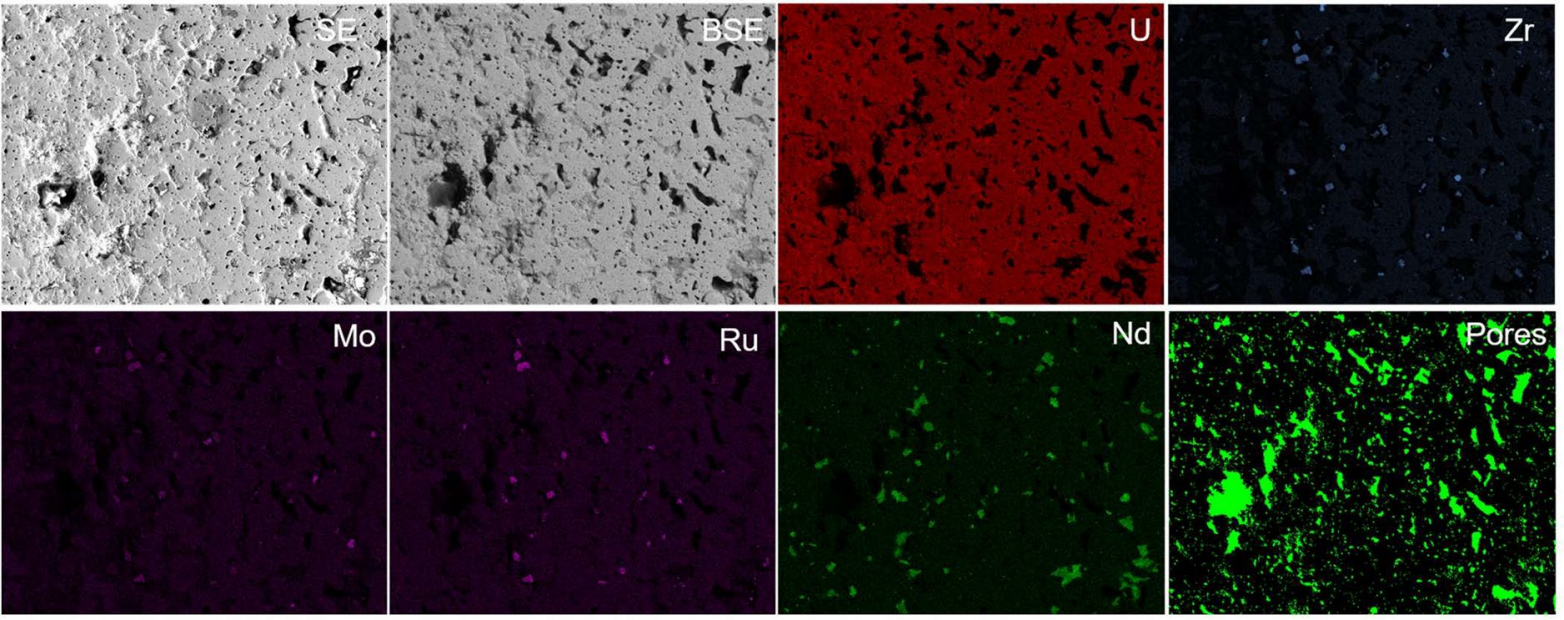
Example of the annotation results based on EDS images.

To design a machine learning model and validate its performance on different image formats and length scales, we prepared the datasets consisting of original images and their corresponding annotated images. Manually annotating the pores on original images can be challenging due to the different appearances of pores on different image formats and magnifications (Fig. 1). To address this, we used SEM/EDS to obtain corresponding chemical element content. As shown in the image of the elements in Fig. 3, the region with dark appearances indicates no selected element existing. In practice, the pore regions are the darkest regions without any chemical element existing. Therefore, the annotation images of pores are generated as follows: 1) using multi-threshold method^38^ to detect the darkest regions (without the corresponding chemical element) on the U, Zr, Mo, Ru, Nd, Fe, Cr EDS images separately; 2) annotating the intersection darkest regions on all EDS images as pores (Fig. 3 pores image). Since it is expensive and labor-intensive to generate images with corresponding EDS images, especially under high magnification settings, we acquired three datasets with a limited amount of data. Firstly, 17 image patches were captured in both SE and BSE formats at 250 × magnification, named SE-250 × and BSE-250 × , respectively. The corresponding annotated results were shared between both image sets. Then, eight SEM images were obtained using BSE format at 2500 × magnification, named SE-2500 × , each with its corresponding annotated image. Each image in the three datasets had a size of 512 × 400 pixels and was captured from different regions of the cross-section images.

### Fine pore-preserving network for pore segmentation

**Figure 4:**
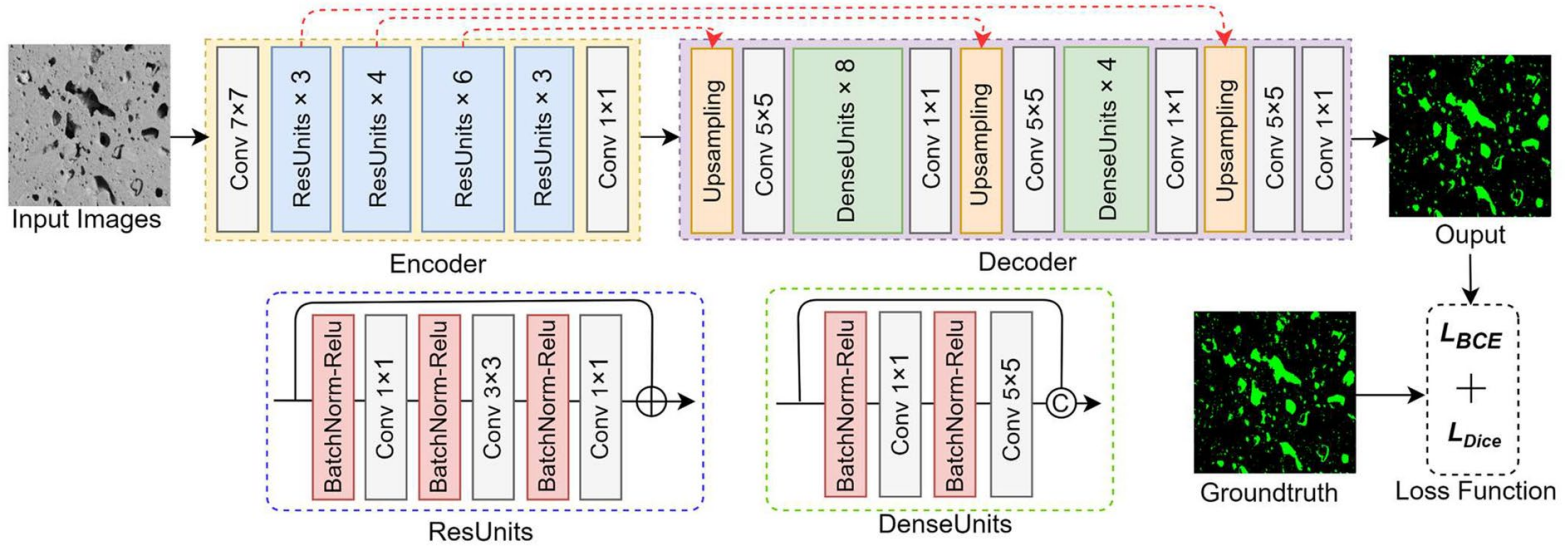
The overview of the proposed neural network architecture.

The proposed network aims to accurately detect and segment the pore foregrounds from the SEM image background. The network tasks the SEM images as input and outputs pore segmentation results represented by pixel-level masks of pore regions. The proposed network architecture is shown in Fig. 4. The proposed network is a fully convolutional deep neural network with an encoder-decoder architecture for accurate pore segmentation. In the encoder, we use ResNet-50^31^ as a feature extractor, which was trained on a public large scale dataset ImageNet^39^, and has achieved advanced and robust performance in many computer vision tasks^32–34^. ResNet-50 is a 50-layer convolutional neural network that contains four residual blocks. Each residual block contains a series of consecutive residual units. Different numbers of residual units are employed in the encoder at different down-sampling stages. Each residual block from downsampling stage 1 to 4 contains 3, 4, 6, and 3 residual units, respectively. The architecture of the residual units is shown in Fig. 4, which contains three consecutive batch normalizations, relu activation layers, and convolutional layers, with a skip connection to connect the gradient flow from the first layer to the last layer.

The decoder comprises a series of up-sampling layers, convolutional layers, and dense units^35^ to reconstruct the encoded latent representation to pore predictions. For the proposed method, multiple dense units are stacked to build a large receptive field with minimal network parameters. Compared to convolution layers with larger kernel sizes, dense units ensure efficient gradient propagation and fewer parameters. Dense blocks are used after the first and second up-sampling operations, with four and eight consecutive dense units, respectively. Our proposed method follows with the U-Net^29^ design, using skip connections to incorporate features from the earlier layers in the encoder to the decoders. The low-level image information is particularly important in the semantic segmentation tasks. Finally, a 1 × 1 convolutional layer with a softmax layer outputs the pore foreground and background. Padding convolution is performed throughout the two upsampling branches to prevent poor predictions at the boundary, resulting in a smaller output size than the input.

#### Learning objective

The learning objective of the proposed network is to segment the pore foreground from the background. The total loss function consists of two terms: Binary Cross-Entropy loss $(L_{BCE}$) and Dice loss $(L_{Dice}$). Let *P* and P* be the predicted pore mask and the ground truth pore mask, respectively. The total loss can be defined as follows:

1 $$ L_{total} \left( {P,P^{*} } \right) = L_{BCE} \left( {P,P^{*} } \right) + L_{Dice} \left( {P,P^{*} } \right) $$

2 $$ L_{BCE} \left( {P,P^{*} } \right) = - \frac{1}{n}\mathop \sum \limits_{i}^{n} P_{i}^{*} \log \left( {P_{i} } \right) $$

3 $$ L_{Dice} \left( {P,P^{*} } \right) = 1 - \frac{{2 \times \mathop \sum \nolimits_{i}^{n} P_{i} P_{i}^{*} }}{{\mathop \sum \nolimits_{i}^{n} P_{i} + \mathop \sum \nolimits_{i}^{n} P_{i}^{*} }} $$

where $P_{i}$ is the categorical class prediction at point *i-th*, and *n* denotes the total number of pixels in the image patch.

## Experimental results

### Dataset and evaluation metrics

#### Dataset

As described in section "Fine pore-preserving network for pore segmentation", we prepared and utilized three datasets for training the proposed model and evaluating its performance. The SE-250 × and BSE-250 × datasets were used for evaluating the network's performance and conducting porosity analysis due to their relatively larger dataset size. The BSE-2500 × dataset was used to evaluate the transfer learning strategies employed in the proposed network.

#### Evaluation metrics

The pixel-level metrics: Recall ratio, Precision, and F1 score were used to evaluate the methods. Recall ratio, Precision, and F1 score are formally defined by

4 $$ {\text{Recall}} = \frac{TP}{{TP + FN}} $$

5 $$ {\text{Precision}} = \frac{TP}{{TP + FP}} $$

6 $$ {\text{F}}1{ }\;{\text{score}} = \frac{TP}{{TP + \frac{1}{2} \left( {FP + FN} \right)}} $$

where TP, FP, FN are the total pixel number of true positive, false positive, and false negative, respectively. The Recall ratio measures the percentage of annotated pores that are accurately detected by the model, while the Precision measures the percentage of predicted pores that are correctly detected. The F1 score is a combination metric of Recall and Precision for measuring the overall detection performance. In the context of pore detection from SEM images, pixel-level metrics are adopted to accurately evaluate the segmentation performance.

#### Implementation details

The proposed network takes the input size of 256 × 256 pixels. Due to the small amount of data, we applied the leave-one-out cross-validation^40^ to evaluate the whole process. In each leave-one-out experiment, one single image is used as a test image, and the rest of the images are employed as the training set for network training. The image augmentation approaches, e.g., random flip, random rotation, Gaussian blur, and median blur, are employed in the training stage. In total, 340 training image patches were generated. The training epoch of the network is set as 100, and the initial learning rate for the Adam optimizer is set as 10^−4^ and is reduced to 10^–5^ after 50 epochs. The batch size is 4 for training the model. The postprocessing morphology operations (e.g., fill the holes, remove the small objects) are employed to generate fine segementation maps.

### The effectiveness of the proposed network

**Figure 5:**
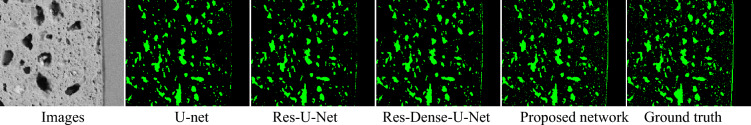
The visual representation of the effectiveness of the network design on BSE-250 × dataset.

The proposed network introduces two major contributions, ResNet blocks^31^ and Dense blocks^35^, compared to the U-Net^29^. To evaluate the effectiveness of these components, we conducted experiments and compared our network to three networks: 1) the U-Net with a ResNet encoder (Res-U-Net), where its decoder architecture is similar to our method but replaces all the Dense block with convolutional layers; 2) the U-Net with a ResNet encoder and a single Dense block in the decoder (ResNet-Dense-U-Net), where its decoder architecture has a similar architecture to our method but replaces the second Dense block with convolutional layers; and 3) the U-Net with a ResNet encoder and two Dense blocks in the decoder (Ours). The experiments were conducted on the BSE-250 × dataset and evaluated using Recall ratio, Precision, and F1 score. As shown in Table 2, with the ResNet block, the Res-U-Net improved the U-Net by 2.9% and 1.3% in Recall ratio and F1 score, respectively. Adding a single Dense block, Res-Dense-U-Net improved the Res-U-Net by 3.9% and 2.1% in Reccall ratio and F1 score, respectively. Our method achieved the best overall performance and improved the U-Net by 11.2% and 4.5% in Recall ratio and F1 score, respectively. Figure 5 provides a sample of the segmented results of the networks, and it is worth noting that the integration of two components into the U-Net led to an improvement in the visual representations. Both quantitative and qualitative results demonstrate that incorporating ResNet blocks and Dense blocks can significantly improve segmentation performance in Recall and F1 score, highlighting the effectiveness of the two major components in our proposed method.

**Table 2:** Effectiveness of the network design.

Networks	Recall	Precision	F1 score
U-Net	0.7349	**0.9390**	0.8232
Res-U-Net	0.7572	0.9321	0.8345
Res-Dense-U-Net	0.7884	0.9285	0.8523
Ours	**0.8282**	0.9012	**0.8624**

Significant values are in [bold].

### Overall performance

To demonstrate the effectiveness of the proposed network, our method was compared with two deep supervised neural networks: SegNet^36^, and U-Net^29^ on pore segmentation. SegNet and U-Net are benchmark fully convolutional networks for semantic image segmentation. Compared to U-Net, our network has two major improvements: 1) we use ResNet units instead of convolutional layers in the encoder to eliminate gradient vanish problems, and 2) we employ dense units instead of single convolutional layers in the decoder to improve the network efficiency with limited resources. Compared to U-Net and our network, SegNet uses the computed pooling indices to transfer knowledge from the encoder to the decoder instead of a skip-connection design to transfer the low-level features to the decoder, but all networks share a similar encoder-decoder design. The experiments were conducted on the BSE-250 × dataset using three metrics: Recall, Precision, and F1 score. Leave-one-out cross-validation was applied to all methods for a fair comparison. The overall results are shown in Table 3.

**Table 3:** Comparisons of the proposed method and state-of-the-art segmentation methods.

	BSE 250 × dataset			SE 250 × dataset		
Method	Recall	Precision	F1-score	Recall	Precision	F1-score
SegNet	0.7265	0.9236	0.8120	0.5331	0.7569	0.6201
U-Net	0.7349	**0.9390**	0.8232	0.4359	**0.8309**	0.5647
Ours	**0.8282**	0.9012	**0.8624**	**0.6873**	0.7433	**0.7105**

Significant values are in [bold].

**Figure 6:**
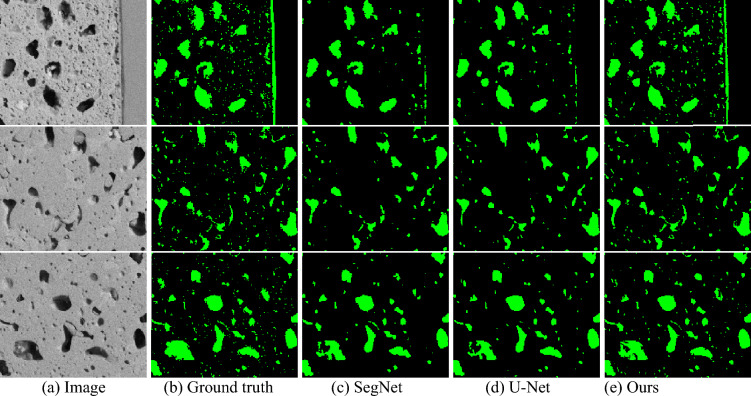
The visual representation of comparison with state-of-the-art methods on BSE-250 × dataset.

For BSE images, our method achieved the best Recall, and the F1 score among all the methods. The Recall value improved by 11.26% compared to the second-best method, U-Net, indicates that our method is capable of outputting more positive predictions. The F1 score shows the overall detection performance, and our method achieved a value of 0.8624, which is promising for pore detection. The Precision score of 0.9012 indicates that the accuracy of positive prediction is reliable. Meanwhile, SegNet and U-Net methods have the Recall score range of 0.72 to 0.74, and the Precision score range of 0.92 to 0.94, indicating that they return fewer predicted labels, but most of the predicted labels are correct. In Fig. 6, the qualitative visual comparison of BSE images shows that SegNet and U-Net return fewer predicted pores compared to the ground truth and our methods (relatively low Recall), especially for small pores’ detection and the pores with irregular shapes.

**Figure 7:**
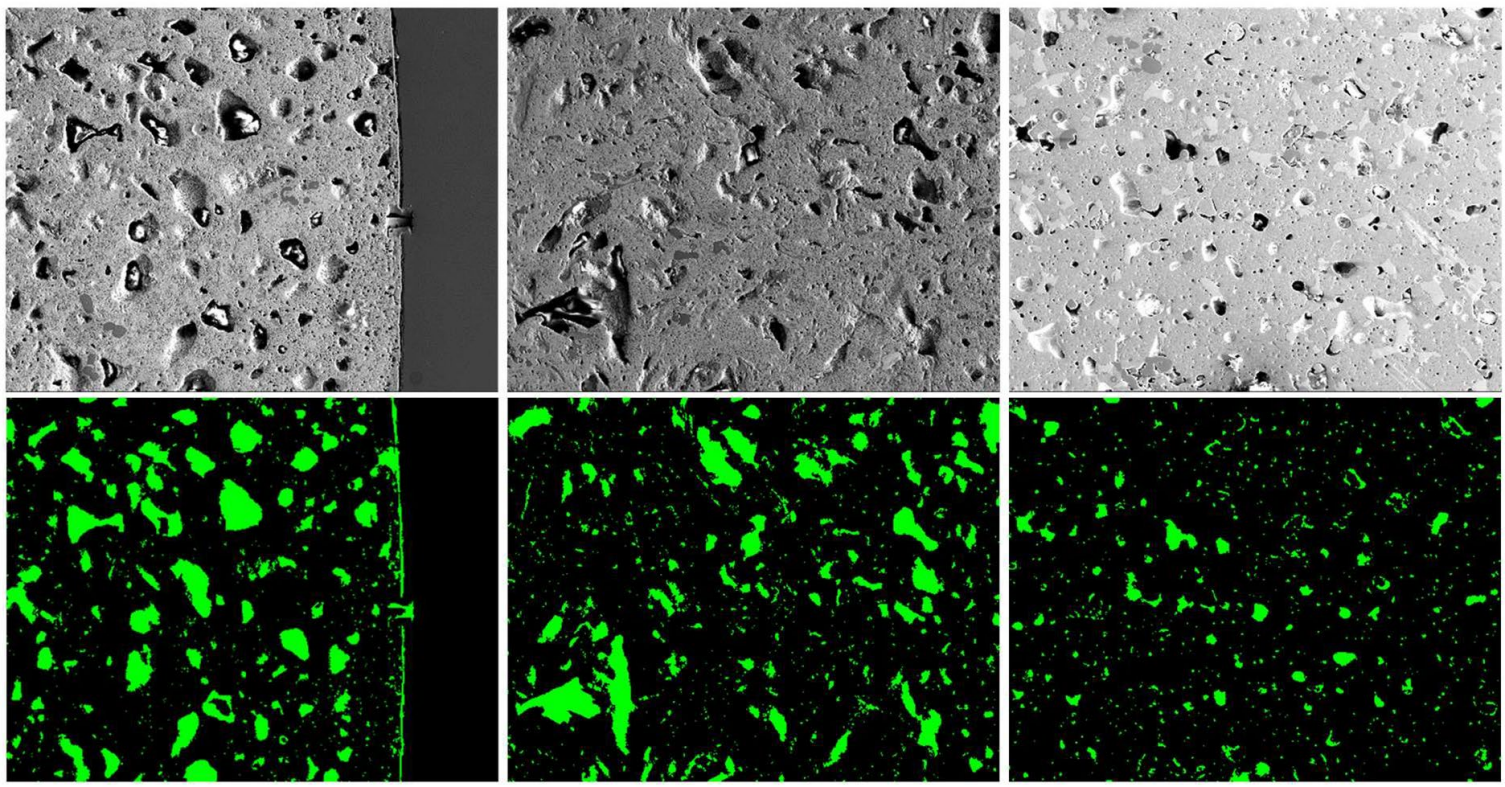
Results of pore segmentation using our method on SE-250 × images.

For SE images, the model was trained and tested using the SE images with the annotated images. The model performance was evaluated using Recall, Precision, and F1 score by conducting leave-one-out cross-validation on the dataset. As shown in Table 3, we achieved the highest Recall and F1 score among all the methods, with Recall, Precision, and F1 score of 0.6873, 0.7433, and 0.7105, respectively. However, compared to the prediction results from the BSE images, the performance of all methods on the SE images was slightly worse on all metrics. This is due to the fact that SE images mainly show the different topography and morphology of the microstructure with less composition information of pores, which may miss part of the microstructures. Therefore, the SE images’ test performance may be slightly worse than the BSE images, but SE images can still be used to detect the pores and achieve relatively reliable performance. Figure 7 shows examples of the segmented pores from SE images.

**Figure 8:**
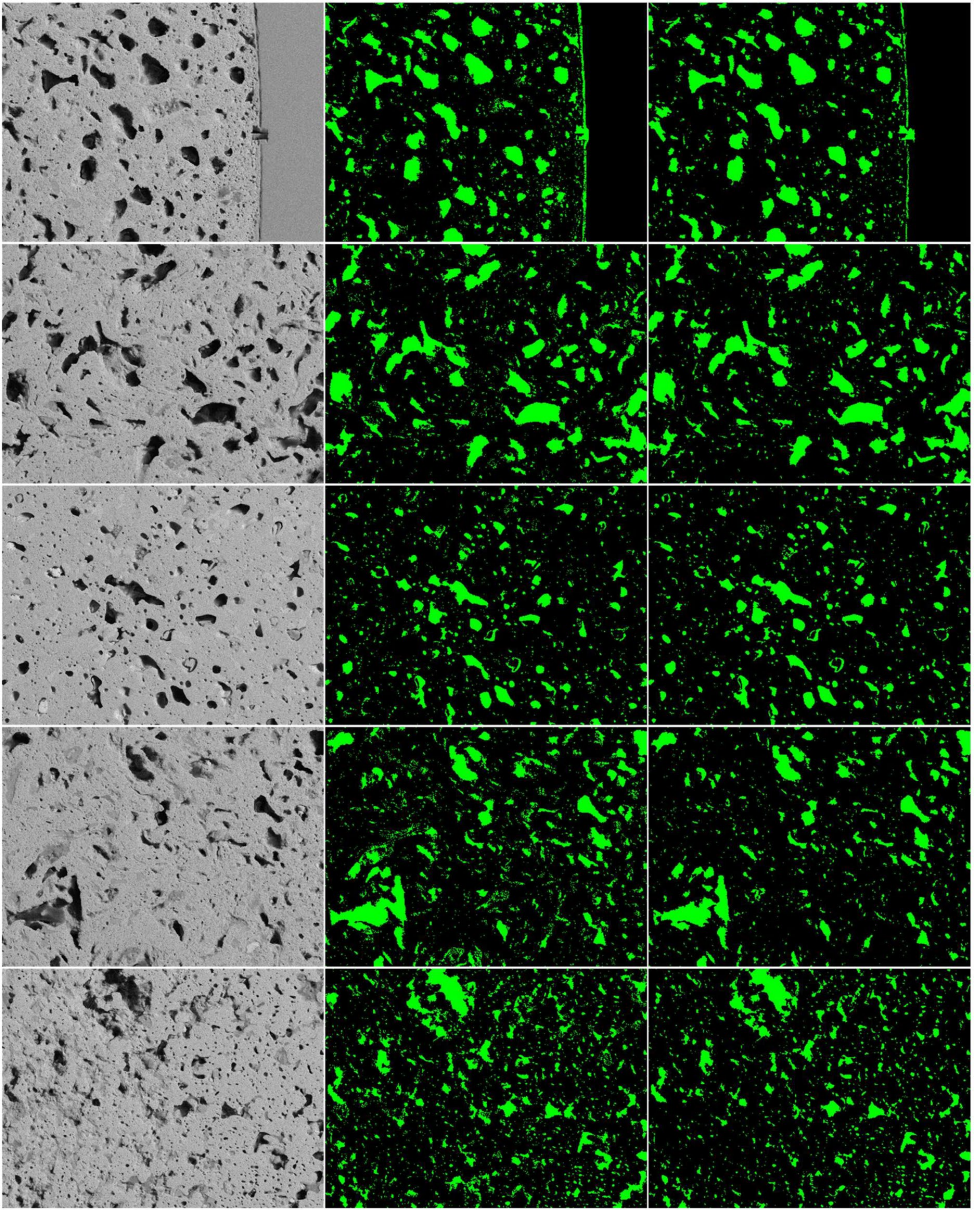
Results of pore segmentation using our method on BSE images. The left column shows the original BSE images; the second column shows the corresponding ground truths; and the right column shows the segmented results. The top three rows display the three images with the highest F1-score, while the bottom two rows show two images with the lowest F1-score.

To show the proposed model is not sensitive to outlier images, we present the leave-one-out cross-validation results of every single test image on BSE images using Recall, Precision and F1 score metrics in Table 4. From the F1 score results, the minimum score is 0.8146; the maximum score is 0.9019; the mean score is 0.862, and the mean is close to the high end of the score range, which suggests that the predictions achieved promising results. The standard deviation score of 0.027 compared to the score range of 0.0873 is relatively small, indicating that the model performed consistently and there is low variability in predictions. Figure 8 shows more predicted examples of BSE images. We present the three best images with the top three highest F1 score (images 1,2,7- [top three row]), and two images with the lowest F1 score presented in Table 4 (images 11, 14- [bottom two]). From the images, we can observe that the three best predictions have close visual representations compared to the ground truths. The two images with the lowest F1 score are close to ground truth in large objects, but face the challenges to detect the small objects. Overall, from the observation of the quantitative and qualitative results, our methods achieved promising pore segmentation performance.

**Table 4:** Results of leave-one-out cross validation on BSE images using Recall, Precision, and F1-score metrics.

Test image	Recall Ratio	Precision	F1 score
1	0.8641	0.9254	0.8937
2	0.8789	0.9262	0.9019
3	0.8303	0.9069	0.8669
4	0.7956	0.8670	0.8297
5	0.7830	0.8771	0.8274
6	0.8333	0.8552	0.8584
7	0.8533	0.9306	0.8903
8	0.8301	0.9270	0.8759
9	0.8274	0.9391	0.8797
10	0.8379	0.9100	0.8725
11	0.7328	0.9170	0.8146
12	0.8270	0.9151	0.8688
13	0.8045	0.8769	0.8392
14	0.7412	0.8978	0.8120
15	0.8947	0.8479	0.8707
16	0.8687	0.8826	0.8756
17	0.8771	0.8893	0.8831
Mean $\pm$ SD	0.828 $\pm$ 0.045	0.901 $\pm$ 0.028	0.862 $\pm$ 0.027

### Small pore detection

**Figure 9:**
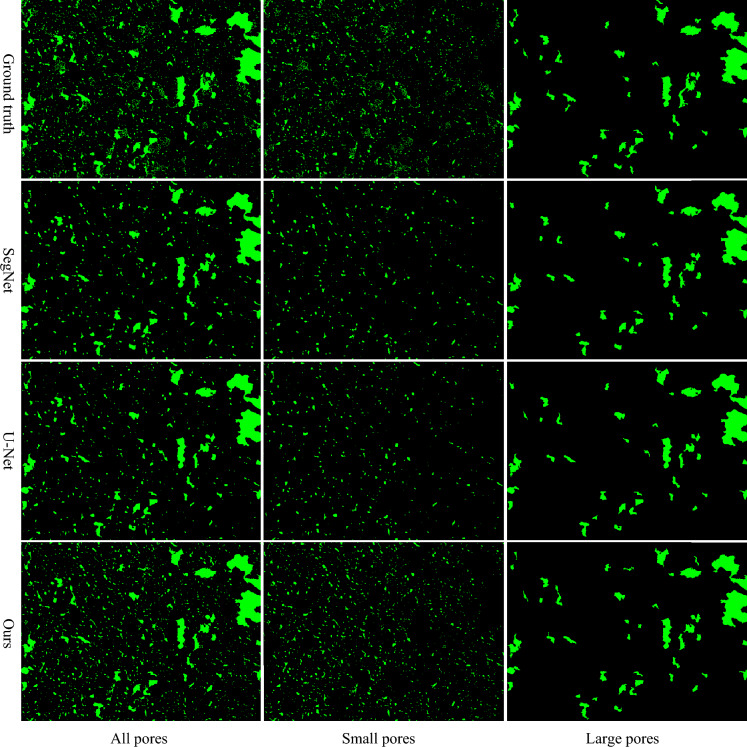
Qualitative comparison of segmented pores with different pore size on BSE-250 × dataset.

Measuring pore size and distribution is crucial for understanding material performance. Detecting small pores is a major challenge in pore segmentation on SEM images. However, current pixel-level evaluation metrics evaluate the performance of the model at individual pixel levels without considering the size of the detected objects. Therefore, a model that performs well in detecting large pores may have difficulties in detecting small pores. In this section, we evaluate the model performance based on different pore sizes, and we categorize pores into small and large pores and evaluate them separately. Let *S* represent the size of each individual pore, where the value of S is the total number of connected pixel points in the object. We categorize pore size into two categories: 1) small pores, where the region contains 50 or fewer pixels, and 2) large pores, where the region contains more than 50 pixels. We used Recall, Precision, and F1 score to evaluate segmentation performance on different sizes of pores, and we evaluated the model on the BSE-250 × image set with the leave-one-out cross-validation experiments. Table 5 shows the results, the proposed method achieved the best Recall and F1 score in small pore detection, improving 27.1%, and 15.4% on Recall and F1 score, respectively, compared to the second-best method (U-Net) in detecting small pores. All the models achieved promising detection performance in detecting large pores, with our proposed method achieving the best overall results. Although the proposed method outperformed SegNet and U-Net in small pore detection, our Recall score of 0.5791 is still limited, and there is room for improvement in the future. Detecting small pores is challenging due to the following major reasons in the BSE images: 1) small pores can be extremely small, appearing as a single pixel in width and height in images, 2) small objects may be dispersedly distributed in the images, and the matching ground truth with BSE images may not have obvious image factors on these small objects. Figure 9 shows an example of a BSE image with different pores size. The second to fourth rows are the predicted results from all models. Our method classified more small pores compared to the SegNet and U-Net methods.

**Table 5:** Quantitative comparison of segmentation results on different pore sizes.

Size	Method	Recall Ratio	Precision	F1 score
Small pores (≤ 50 pixels)	SegNet	0.4153	0.7272	0.5273
	U-Net	0.4225	**0.7842**	0.5471
	**Ours**	**0.5791**	0.7396	**0.6471**
Large pores (> 50 pixels)	SegNet	0.8716	0.9260	0.8975
	U-Net	0.8999	**0.9439**	0.9072
	**Ours**	**0.9026**	0.9190	**0.9105**

Significant values are in [bold].

### Application to 2500 × magnifications

**Figure 10:**
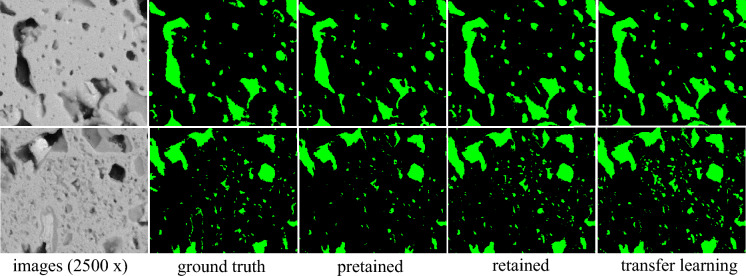
Qualitative results on BSE-2500 × dataset.

High magnification images can provide detailed information about the sample, but it is more challenging to prepare the high-power field images with the corresponding ground truth, and those data size is limited. To solve the limited data issue in high magnification images, training, testing, and transferring the model at lower magnifications can help to assess the generalizability of the model to different magnifications. To evaluate the performance of the proposed network at different magnifications, we conducted three experiments: 1) directly applied the pre-trained network (trained at BSE-250 × magnification) to images at BSE-2500 × magnification; 2) retrained and updated the network with the BSE-2500 × images; 3) employed transfer learning to transfer the existing knowledge from a larger dataset of images at 250 × magnification to a smaller dataset of limited images at 2500 × . Specifically, we utilized the pre-trained weight on the BSE-250 × images to retrain the limit images at BSE-2500 × . The experimental results are presented in Table 6, and the qualitative results are shown in Fig. 10. When directly applied to images at 2500 × , the pre-trained network achieved an F1-score of 0.7422 and the qualitative results showed reliable pore detection performance. Retraining our network with the new 2500 × images resulted in a Recall, Precision, and F1-score of 0.7905, 0.8313, and 0.8001, respectively. The transfer learning approach, integrated with the retrained model, achieved a Recall, Precision, and F1-score of 0.8197, 0.7888, and 0.8132, respectively, outperforming the other two experimental strategies. The results suggest that the transfer learning approach integrated with the retrained model achieves the best performance among the three experimental strategies.

**Table 6:** Quantitative results on BSE-2500 × dataset.

Experiments	Recall Ratio	Precision	F1 score
Pretrain-using 250 ×	0.6476	**0.9262**	0.7422
Retrain-using 2500 ×	0.7905	0.8313	0.8001
Transfer learning-retrain	**0.8197**	0.7888	**0.8132**

Significant values are in [bold].

### Porosity analysis using the fuel cross-sectional imaging

In this section, we conducted porosity analysis on the fuel cross-sectional images. In Section "Material and data collection", we discussed the details of the preparation of the cross-sectional images. The experiments were conducted on SE cross-sectional images. Although BSE images are preferred for pore analysis because they reveal the morphology of pores, due to the unavailability of BSE cross-sectional images, SE images were used instead. The pore regions in each SE image patch were predicted by utilizing the pre-trained proposed network on the SE-250 × dataset and then concatenated the patches to form cross-sectional images with the predicted pores.

**Figure 11:**
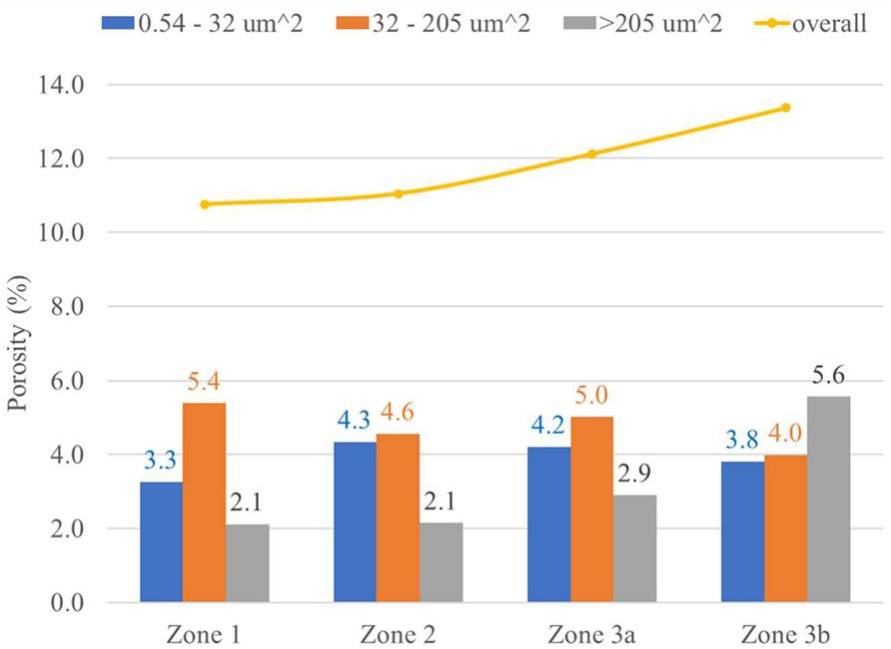
Analysis of porosity in various zones of the cross-sectional imaging.

Figure 2a presents an example of the fuel cross-sectional image. In a fuel cross-sectional image, there are three major zones based on the Zr content^12^ described the details zone categorization in a post-irradiation examination (PIE) work through advanced characterization. As shown in Fig. 2a, Zone 1 is the fuel central region up to 0.32 R (R—fuel radius after irradiation), with enriched Zr content. Zone 2 is the Zr depleted region approximately 0.32 R to 0.48 R. Zone 3 has intermediate Zr content, with Zr content slightly higher than the as-fabricated condition. Zone 3 can be divided into Zone 3a (0.48 R to 0.63 R) and Zone 3b, the latter of which is in the fuel peripheral region with higher Zr content than the former. Though the Zr content stays relatively the same within each zone, each zone has multiple crystallographic phases^12^. The as-fabricated fuel pin radius is 2.49 mm^8^. The radius of the irradiated fuel cross-section is about 2.875 mm, filling the gap between the fuel pin and the cladding. The overall porosity measured from the fuel cross-section is about 12.6% (pore area/the area of the fuel cross-section), accounting for about half of the fuel radial swelling. It is important to note that due to the SEM resolution, any pore smaller than 0.54 µm^2^ is not detectable. The zone porosity increases from center (Zone 1) to the fuel peripheral region (Zone 3b) as shown in Fig. 11. The pores are divided into three categories based on their sizes. The porosity contribution from small pores (area between 0.54 and 32 µm^2^, corresponding to diameter 0.8 and 6.4 µm in which $diameter=2\times \sqrt{area/\pi }$ ) increases from 3.3% in Zone 1 to 4.3% in Zone 2, stays relative the same (4.2%) in Zone 3a, and decreases to 3.8% in Zone 3b. The porosity contributed from the intermediate-sized pores (32 < area < 205 µm^2^ or equivalent 6.4 < diameter ≤ 16.2 µm of round-shaped pores) scatters between 4% and 5.4% without a clear trend. The large-sized pores (area coverage > 205 µm^2^, corresponding to diameter > 16.2 µm of round-shaped pores) take 2.1% of the porosity in both Zones 1 and 2 and increase to 2.9% and 5.6% porosity in Zone 3a and 3b.

**Figure 12:**
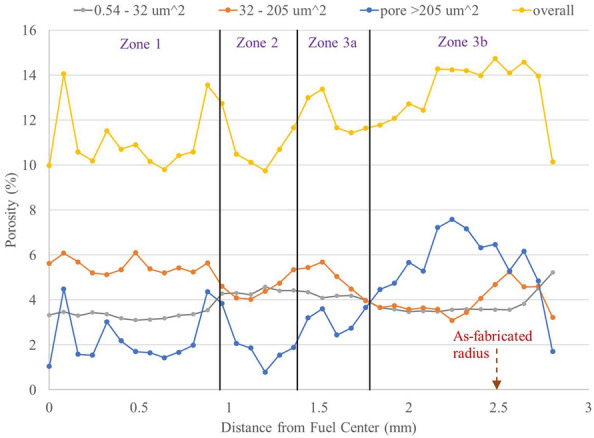
The porosity distribution (including overall porosity as well as the porosity contribution from three different size pores) as a function of the distance from the fuel center. The vertical black lines are the zone boundaries. The as-fabricated fuel radius (before irradiation) is also labelled.

To further study the localized porosity distribution, the fuel cross-section is separated into 36 concentric annuli with each annular thickness approximately 0.08 mm, and the porosity for each annular, including overall porosity in the annulus as well as the porosities contributed from three pore size, is shown in Fig. 12. The porosity from small pores (0.54–32 µm^2^) stays relatively constant (3.1%–3.3%) in Zone 1 and has a step increase to 4.3% near the boundary of Zone 1 and Zone 2. It remains flat in Zones 2 and 3a but decreases slightly to 3.5% after the boundary of Zones 3a and 3b. It maintains around 3.5% in Zone 3b until close to the fuel peripheral region, where it starts to increase up to 5.2%. On the other hand, both porosities from intermediate and large pores fluctuate in Zone 1. They have a “U”-shape trend in Zone 2. Zone 1 has multiple crystallographic phases, three major phases and up to five sub-phases according to TEM, while Zone 2 has been observed mainly as an α-U matrix with Zr nano-precipitates^12^. Multiple crystallographic phases may create more phase boundaries and grain boundaries, which can serve as a sink for the fission gas, resulting in pore growth. Since multiple crystallographic phases are randomly distributed in Zone 1, it is not surprising to see the fluctuations of the distribution of intermediate and large pores. Zone 2 has more intermediate and large pores near the boundaries with Zone 1 and Zone 3a than that of the zone center. In Zones 3a and 3b, the porosity from intermediate pores seems to follow an opposite trending of the porosity from the large pores, except near the fuel peripheral region, where porosity from both the intermediate and large pores decrease around as-fabricated radius to cladding but porosity from the small pores increases at the regions. The porosity from large pores reaches maximum near the center of Zone 3b but decreases when approaching the fuel edge. A similar trend is observed for U-10Zr annular fuel, where large pores decrease the contribution to porosity when moving closer to the inner cladding surface^25^. The less large pores near the fuel edge may reduce the FCCI, as the large pores can provide a pathway for lanthanides to reach and react with the cladding. The reason why small pores increase but large pores decrease is not clear. Perhaps lower temperatures and certain crystallographic phases at the fuel edge reduce fission gas diffusion, so that the pores grow slowly. For example, the γ2 phase (a Zr-rich body centered cubic solid solution between U and Zr) is surprisingly present at Zone 3b^12^. The γ2 seems to have low Xe (one type of fission gas) diffusivity^41^.

## Conclusion

In this paper, we first introduced a data processing workflow for generating multi-source SEM image data to understand the metallic fuel. We proposed a deep fully convolutional network with the residual units and the dense units for accurate pore segmentation and small pore detection. We evaluated our network using Recall, Precision, F1 score metrics on two SEM image datasets. The experimental results demonstrated that our method outperforms two recent fully convolutional networks both quantitatively and qualitatively. Additionally, the generated visual representation results exhibit the effectiveness of our method. We further trained and tested the proposed method on SEM images under different magnifications and showed that transfer learning improves the model performance by transferring the existing knowledge into new SEM images with various magnifications. Finally, we use the pre-trained network to predict SEM images in a whole cross-sectional fuel, and statistical analysis of the predicted pore distribution in the fuel bridges the gap between fundamental understanding and practical application. Overall, this paper proposes a comprehensive pipeline for conducting porosity analysis to study U-10Zr metallic fuel. We propose an accurate and robust deep neural network that significantly improves pore segmentation performance.

The use of machine learning provides us with unprecedented quantitative data on pore distribution and characteristics. How these pore statistics affect fuel performance is still under study, and these data are aimed to support the development of fuel modeling tools (such as BISON). Currently, the ongoing work is focused on crystallographic phase identification quantitatively combining SEM–EDS, TEM, and machine learning.

## Data Availability

The datasets generated and/or analyzed during the current study are not publicly available due to the laboratory policy but should be available 3–5 years after the article is released. Currently, partial data is available from the corresponding author on reasonable request.
